# Gut Microbiome Alterations in Mild Cognitive Impairment: Findings from the ALBION Greek Cohort

**DOI:** 10.3390/microorganisms13092112

**Published:** 2025-09-10

**Authors:** Konstantinos Rouskas, Eirini Mamalaki, Eva Ntanasi, Marianna Pantoura, Maria Anezaki, Christina Emmanouil, Nil Novau-Ferré, Mònica Bulló, Antigone S. Dimas, Christopher Papandreou, Mary Yannakoulia, Anagnostis Argiriou, Nikolaos Scarmeas

**Affiliations:** 1 Institute of Applied Biosciences, Centre for Research & Technology Hellas, 57001 Thessaloniki, Greece; rouskas@certh.gr (K.R.); mpantoyra@certh.gr (M.P.); argiriou@certh.gr (A.A.); 2 1st Department of Neurology, Aiginition Hospital, National and Kapodistrian University of Athens Medical School, 11528 Athens, Greece; eir.mamalaki@gmail.com (E.M.); e.ntanasi@hotmail.com (E.N.); 3 Institute for Bioinnovation, Biomedical Sciences Research Center ‘Alexander Fleming’, Fleming 34, 16672 Vari, Greece; anezaki@fleming.gr (M.A.); emmanouil@fleming.gr (C.E.); dimas@fleming.gr (A.S.D.); 4 Nutrition and Metabolic Health Research Group (NuMeH), Department of Biochemistry and Biotechnology, Rovira i Virgili University (URV), 43201 Reus, Spain; nil.novau@urv.cat (N.N.-F.); monica.bullo@urv.cat (M.B.); 5 Institute of Health Pere Virgili (IISPV), 43204 Reus, Spain; 6 Center of Environmental, Food and Toxicological Technology—TecnATox, Rovira i Virgili University, 43201 Reus, Spain; 7 CIBER Physiology of Obesity and Nutrition (CIBEROBN), Carlos III Health Institute, 28029 Madrid, Spain; 8 Department of Nutrition and Dietetics Sciences, School of Health Sciences, Hellenic Mediterranean University, 72300 Siteia, Greece; papchris10@gmail.com; 9 Clinical and Epidemiological Neuroscience (NeuroÈpia), Institut d’Investigació Sanitària Pere Virgili (IISPV), 43204 Reus, Spain; 10 Department of Nutrition and Dietetics, Harokopio University, 17671 Athens, Greece; myianna@hua.gr; 11 Department of Food Science and Nutrition, University of the Aegean, Myrina, 81400 Lemnos, Greece; 12 The Gertrude H. Sergievsky Center, Department of Neurology, Taub Institute for Research in Alzheimer’s Disease and the Aging Brain, Columbia University, New York, NY 10032, USA

**Keywords:** gut microbiota, microbiome, mild cognitive impairment, Alzheimer’s disease, discrimination model, microbiota-gut–brain axis

## Abstract

Emerging evidence suggests a potential role of gut dysbiosis in neurodegenerative disorders and, in particular, Alzheimer’s disease (AD) pathology and cognitive decline. However, the role of gut microbiome in the early prodromal stages of AD and particularly in mild cognitive impairment (MCI) remains understudied and has been mostly explored in Asian populations with no representation of European populations. To address this research gap in the literature and to suggest novel microbiome features associated with MCI, we conducted a cross-sectional study in a European population sample and profiled gut microbiota in 99 individuals without dementia through 16s ribosomal RNA (rRNA) sequencing. Individuals were categorized by cognitive status based on standard clinical criteria to cognitively normal (n = 49) or individuals with MCI (n = 50). Differential abundance through Microbiome Multivariable Associations with Linear model (MaAsLin2) and elastic net logistic regression analyses were used to identify gut microbiome features associated with MCI. MCI group was older than the CN group and age was used as covariate in the differential abundance analysis. No differences in alpha and beta diversity were found between the two groups (*p* > 0.05). At false discovery rate (FDR) < 0.05, we identified specific genera associated with MCI, mostly linked to short chain fatty acids (SCFAs) production (e.g., *Candidatus_Soleaferrea q* = 0.027, MaAsLin2 coefficient = 1.65, *Sellimonas q* = 0.017, MaAsLin2 coefficient = −4.45), while we highlight nominal (*p* < 0.05, *q* > 0.05) correlations of genera (e.g., *Hydrogenoanaerobacterium*, *Subdoligranulum*) with metrics of cognitive assessment. Microbiota was shown to have a fairly good discriminative capacity for MCI status (area under the curve AUC = 0.77), with *Rothia* genus found as the top predictor for MCI (beta coefficient [95% confidence intervals] = 0.224 [0.216–0.233]). Overall, our findings add to current knowledge reporting gut microbiome alterations in MCI by suggesting novel associated microbiome features; however, larger scale longitudinal studies are needed to further elucidate the underlying biological pathways linked to the disease.

## 1. Introduction

Alzheimer’s disease (AD), the leading cause of dementia worldwide, is a complex neurodegenerative disorder with substantial societal and economic impact [1]. Its pathology follows a continuum that begins from the preclinical stage, marked by normal cognition but underlying amyloid-β deposition, tau aggregation, neuroinflammation and neurodegeneration [2], and progresses to mild cognitive impairment (MCI) and then to clinically apparent dementia. The preclinical events of AD can occur over a decade before symptoms appear [3,4,5], highlighting the importance of early diagnosis [6]. MCI is an intermediate phase of the AD continuum between the normal brain aging and dementia [7,8], marked by noticeable deficits in various cognitive functions including memory, executive function, attention, language, and visuospatial skills [9]. MCI is more frequent in older people with its prevalence increasing with age [10], while the diagnosis relies on clinical and neuropsychological assessment accompanied with international guidelines [9]. Although some individuals with MCI may revert to normal cognitive status within 1–3 years from their diagnosis [11], evidence consistently reports that individuals with MCI are at higher risk of progressing to dementia compared to age-matched controls [10]. Given this heterogeneity in the nature of MCI, it is suggested that various factors such as brain pathologies and systemic inflammation play a role in disease progression [12]. Therefore, it is crucial to identify novel specific less-invasive markers to predict the progression from MCI to dementia and to understand the biological changes in this stage, thus, enabling research community design-targeted interventions aiming ultimately to the delay of the onset of dementia.

Accumulating evidence suggests that gut microbial dysbiosis is involved in neurodegenerative diseases [13] and particularly in AD pathogenesis via the microbiota-gut–brain axis and by regulating peripheral and central inflammation through microbial metabolites [13,14,15,16,17,18,19,20,21]. Recently, transplants of gut microbiota from patients with AD were shown to induce memory impairments in young mice [22], in line with previous report discussing the loss of cholinergic functions in hippocampal neurons through the microbiota-gut–brain axis [14]. In the field of MCI, evidence regarding the role of gut microbiome remains still sparse with quite controversial findings among studies. The majority of existing studies were conducted in countries from Asia (China, Japan, Republic of Korea) and in a lesser degree from USA. A recent review summarized the few existing studies that had focused on the association of gut microbiome composition with MCI, including 13 observational studies, 1 randomized controlled trial (RCT) and 2 meta-analyses [23]. Most studies concluded an increase in pro-inflammatory Bacteroidetes and *Bacteroides* and a decline in protective Firmicutes and *Faecalibacterium* in MCI subjects compared to healthy controls, while others showed a decrease in *Bacteroides* species in MCI. Towards this direction, a recent metagenomics study in Taiwanese individuals reported additional novel MCI-associated species such as *Ruminococcus* and *Akkermansia* [24], while another study in Chinese defined stage-specific microbiome profiles in the AD continuum including MCI [17]. Overall, current knowledge cannot suggest a core MCI-associated gut microbiome profile and thus more research is needed to elucidate the contribution of gut microbiome in MCI.

Lack of a unique consensus and differences in the associated genera across studies may reflect the heterogeneity of studied MCI populations or the impact of various internal and environmental factors. The composition of the gut microbiome and its diversity is influenced by many factors including diet, lifestyle [25], but also ethnicity [26]. Recently, a large-scale collection of gut microbiome data from over 168,000 participants revealed global microbiome differences across different world regions [27], highlighting the vital importance of studying microbiomes in diverse populations. To our knowledge, to date, most studies investigating the role of the gut microbiome in MCI have focused on participants from Asia [17,28,29] with no representation from European populations. In Europe, Greece is considered one of the fastest-aging countries with the prevalence of dementia projected to nearly triple by 2050 [30]. This demographic trend combined with the growing evidence that distinct environmental factors such as the Mediterranean diet [31] may affect microbiome findings highlights the importance of investigating the gut microbiome in MCI in an underexplored population. Exploring European populations living in a likely different environmental context could enable us to address the current knowledge gap by identifying additional microbiome features associated with MCI and ultimately reveal novel early markers of AD pathology.

Given that the current literature exploring the relationship of gut microbiome with MCI is mostly focused in Asian populations, we conducted a cross-sectional study in a Greek cohort of individuals without dementia and performed gut microbiome profiling through amplicon sequencing. Our aim was to identify novel gut microbial alterations associated with MCI and to evaluate their capacity to discriminate cognitively normal individuals from individuals with MCI.

## 2. Materials and Methods

### 2.1. Study Design and Population Sample

This research has been conducted in the context of the ongoing Aiginition Longitudinal Biomarker Investigation of Neurodegeneration (ALBION) population-based cohort study. This study consists of individuals without dementia aged >40 years old, referred to the Cognitive Disorders Clinic of Aiginition Hospital, affiliated with the National and Kapodistrian University of Athens. The study’s ongoing primary goal is to perform research pertaining to the preclinical and prodromal stages of AD. A detailed description of the ALBION study protocol can be found elsewhere [32,33]. In brief, neurologists conducted a comprehensive interview and clinical examination of study participants. The exclusion criteria were as follows: (1) diagnosis of dementia at baseline [34,35], (2) presence of medical conditions associated with a high risk of cognitive impairment or dementia (including Parkinson’s disease, multiple sclerosis, hydrocephalus, epilepsy, Huntington’s disease, Down syndrome, active alcohol or drug abuse or major psychiatric conditions such as major depressive disorder, schizophrenia, and bipolar disorder), (3) current serious gastrointestinal (GI) disorders that could affect gut microbiome including cancers and inflammatory bowel disease, (4) pregnant or breast-feeding, and (5) taking any antibiotics within three months prior to fecal sampling. In the present study, we included ALBION participants who had available fecal samples in their baseline evaluation and who had also screened for MCI diagnosis (n = 99). Written informed consent was obtained from all participants, and study procedures were approved by the Institutional Review Board and Ethics Committee of the Aiginition University Hospital, National and Kapodistrian University of Athens, Greece (Approval Number: Protocol code: 255, AΔA: ΨΘ6Κ46Ψ8Ν2-8HΩ, date of approval: 10 May 2022, Issuing body: Aiginition University Hospital, National and Kapodistrian University of Athens, Greece).

### 2.2. Clinical Assessment

All participants underwent comprehensive neuropsychological assessment by trained clinical neurologists. Global cognition was assessed using the Mini Mental State Examination (MMSE) [36] as well as the Revised Addenbrooke’s Cognitive Examination (ACE) [37]. A global neuropsychological composite z-score derived from the outcomes of individual cognitive domain scores (memory, language, attention-speed, executive and visuospatial functioning) was also calculated, as previously described [38]. A neurological examination was performed by a specialist neurologist who recorded detailed information regarding demographics, medical history, medication, and family history. Further information on the neuropsychological evaluation in ALBION study can be found elsewhere [32]. Diagnosis of MCI was performed using standard criteria [9,39] and participants were classified into cognitively normal (n = 49) or MCI (n = 50) groups. A detailed schematic showing the study’s workflow is presented in Figure S1.

### 2.3. APOEε4 Status

*APOE*ε*4* genotyping procedures have been described previously [40]. Briefly, *APOE*ε*4* genotyping was performed using a commercial kit (LightMix TIB MOLBIOL, Berlin, Germany) in Roche Light Cycler 2 (Roche diagnostics, Mannheim, Germany apparatus and hybridization probe method. Participants were classified as *APOE*ε*4* carriers (at least one copy of the *APOE*ε*4* gene) and *APOE*ε*4* non-carriers (no copies of the *APOEε4* gene).

### 2.4. Fecal Sample Collection, DNA Extraction, Sequencing, and Pre-Processing

Participants received a sterile fecal collection container and were instructed to collect fecal samples at home. Fecal samples were transported to the hospital in a cooling bag the day following collection and immediately stored at −80 °C. Microbial DNA extraction was performed using two different extraction kits due to differences in the existing infrastructure in collaborating institutes. The first fifty samples were extracted in the Biomedical Sciences Research Center ‘Alexander Fleming’ using the QIAamp DNA Stool Mini Kit (Qiagen, Athens, Greece) and the remaining forty-nine samples were extracted in the Institute of Applied Biosciences, Centre for Research & Technology Hellas using the MGIEasy Stool Microbiome DNA Extraction Kit (MGI Tech, Marupe, Latvia). Sequencing of V3–V4 regions of the 16s ribosomal RNA (rRNA) gene (~460 bp) was performed at the Sequencing and Genomics Facility of CERTH-INAB in Thessaloniki. Libraries were sequenced on Illumina systems MiSeq (Illumina, San Diego, CA, USA) (50 samples extracted with the QIAamp kit; CN = 30, MCI = 20) or NextSeq2000 (Illumina, San Diego, CA, USA) (49 samples extracted with the MGIEasy kit; CN = 19, MCI = 30) (2 × 300 bp paired-end reads). We adjusted our analyses for the sequencing platform to account for the existence of two different sequencing processes and potential batch effects due to use of different extraction kits. Sequencing reads were quality controlled using FastQC v12 [41] and MultiQC v1.27 [42] and denoised into amplicon sequence variants (ASVs) using the DADA2 package [43]. Taxonomy was assigned to ASVs using the SILVA 138 16S rRNA database [44]. Sequences classified as archaeal, chloroplastic or mitochondrial were removed yielding 4142 ASVs for downstream analyses.

### 2.5. Statistical Analysis

Differences in demographics or clinical covariates between the cognitively normal individuals and those having MCI were determined using parametric Welch’s *t*-test for normally distributed data, using non-parametric Mann–Whitney U test for non-normal data and using Fisher’s exact test for categorical data. Statistical significance was set at *p*-value < 0.05.

### 2.6. Alpha and Beta Diversity

We rarefied the ASV table to the lowest sequencing depth (5216 reads, function *rarefy_even_depth*) to account for differential sequencing effort. We calculated four alpha diversity measures (observed richness, Pielou’s evenness, Inverse Simpson and Shannon) using the R package microbiome [45] (function *alpha*). Significant differences between groups were tested using the Wilcoxon test (*p*-value < 0.05). Beta diversity was estimated by using Bray–Curtis dissimilarity. Permutational multivariate analysis of variance (PERMANOVA) was performed using the R package vegan v2.6-4 (function *adonis2*) to calculate variance explained by each study factor and to investigate the microbial community differences between comparison groups using 999 permutations [46]. All PERMANOVA *p*-values were corrected for multiple comparisons using false discovery rate (FDR) and results were plotted by principal coordinate analysis (PCoA).

### 2.7. Differential Abundance Analysis of Genera and of Pathways

To identify differentially abundant (DA) genera among diagnosis groups, we aggregated the ASVs to genus level and only genera with prevalence > 10% and abundance > 0.0001% were tested. Rarefaction to minimum sequencing depth was performed before running the model. Unassigned genera or those assigned as ‘uncultured’ were removed, resulting in 139 genera. Given the compositionality of microbiome data and the potential influence of confounding factors, we applied Microbiome Multivariable Associations with Linear model (MaAsLin2 v1.12.0) [47] to detect microbial signatures associated with MCI. As gut microbiome data are often zero-inflated, we fitted a zero-inflated negative binomial (ZINB) model in the rarefied abundance data and used the selected covariates (age, sex, medication history of hypertension, sequencing platform, and MMSE) as fixed effects (Supplementary Text S1). Particularly for age, differences between the two groups are in line with the current literature highlighting age as a critical factor affecting MCI [17,24,40,48]. In our study, we randomly selected our study participants and to account for age differences between groups, and we adjusted our downstream analyses for age, thus making the groups’ comparison more accurate. Considering that the input data were rarefied, we turned off the default normalization and transformation methods implemented in the MaAsLin2 function. *p*-values were corrected for multiple testing to avoid false positives using the Benjamini–Hochberg method (adjusted *p*-value < 0.05).

To profile functional differences in the microbial community between the two groups, we filtered ASVs for prevalence and abundance as described above and used the Phylogenetic Investigation of Communities by Reconstruction of Unobserved States 2 (PICRUSt2) [49]. We next filtered the predicted MetaCyc pathways for prevalence (>10%) and differences in pathways abundance between groups were tested using Maaslin2, as described above, with a negative binomial (NEGBIN) model.

### 2.8. Correlation Between Bacterial Abundance and Covariates

To explore the relationships between key microbial features and pathways identified via Maaslin2 and clinical covariates, we performed Spearman correlation analysis. Correlation coefficients were calculated using the function *cor.test* of the ‘stats’ package and considered statistically significant at *p*-value < 0.05. We corrected for multiple testing using the Benjamini–Hochberg method (adjusted *p*-value < 0.05). The correlation heatmap was visualized using the package ggcorrplot (v.0.1.4.1).

### 2.9. Discriminatory Capacity of Genera

To explore the discriminative performance of gut microbiota for MCI, we regressed binary variables (MCI) against the 139 identified genera. Due to the high dimensionality, sparsity and multicollinearity of microbiome data, we applied an elastic net regularized logistic regression on centered log-ratio (CLR) transformed counts [50]. This method can perform both feature selection and regularization during model training and can often identify a small number of highly relevant features that capture the underlying patterns in the data. To evaluate the model performance, we obtained a 2 × 2 confusion matrix using the test dataset and calculated true positive, true negative, false positive, and false negative findings using predicted and observed classes. Then, we calculated and adopted three metrics for the model’s performance evaluation: accuracy, F1-score, and area under the curve (AUC), using true positive rate and false positive rate (FPR) [51,52]. Further information is provided in Supplementary Text S2.

## 3. Results

### 3.1. Participant Characteristics and Global Differences in the Gut Microbiome

We first examined the demographic and cognitive profiles of participants (Table 1). The overall sample included 99 individuals aged 65.4 ± 9.5 years, of whom 65 (65.7%) were female. A total of 49 of the 99 individuals were classified as cognitively normal. As expected, individuals with MCI were older and were more likely to have hypertension. In regard to neurocognitive assessment, MMSE and ACE scores were lower in the MCI group compared to cognitively normal individuals, as previously shown [17,28,40]. With regard to the cognitive z-scores, there was a statistically significant difference between the normal and MCI groups in composite z-score and all cognitive domains (memory, attention, language, and executive functioning) except of visuospatial perception. MCI and CN groups did not show any significant difference in sex distribution, body mass index (BMI), *APOE*ε*4* positive status, smoking status, alcohol consumption, and medical history of diabetes and dyslipidemia.

**Figure 1:**
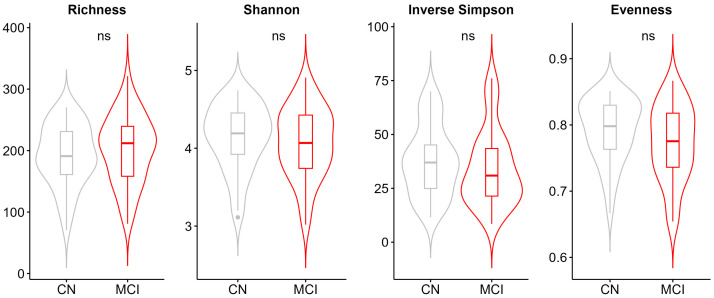
Alpha diversity metrics for the comparison of cognitively normal and individuals with MCI. Alpha-diversity was assessed through four indices which reflect the richness (Observed richness), balance (Pielou’s evenness), and diversity (Shannon, Inverse Simpson’s) of the bacterial community. Abbreviations: MCI; mild cognitive impairment, CN: cognitively normal, ns; not significant.

We then profiled the gut microbiota through 16s rRNA amplicon sequencing and generated a total of 4,981,960 high-quality reads with an average of 50,322 reads per sample. At phylum level, the bacterial community in both groups was dominated by the phyla Firmicutes and Bacteroidetes, which account for 61% and 31% of the total community abundance, respectively (Figure S2A). The Firmicutes/Bacteroidetes ratio did not differ between the cognitively normal and individuals with MCI (mean [SD], CN 2.43 [1.53]; MCI 2.61 [1.92], *p* = 0.95). The three predominant bacterial families were *Lachnospiraceae*, *Bacteroidaceae*, and *Ruminococcaceae* without any significant difference between groups (Figure S2B). Among the top 10 abundant genera (Figure S2C), *Faecalibacterium* (relative abundance CN: 9.1% vs. MCI: 7.5%, *p* = 0.033)*, Blautia* (relative abundance CN: 4% vs. MCI: 2%, *p* = 0.012), *Subdoligranulum* (relative abundance CN: 2.7% vs. MCI: 1.8%, *p* = 0.028), and *Eubacterium_coprostanoligenes_group* (relative abundance CN: 1.7% vs. MCI: 2.6%, *p* = 0.042) showed a statistically significant difference between groups. No difference was found for genus *Bacteroides* in contrary to a recent review supporting increased abundance of this genus in individuals with MCI [23]. In terms of alpha (within-sample) diversity, we did not find significant differences between the cognitively normal individuals and individuals with MCI (Figure 1 and Table S1).

**Figure 2:**
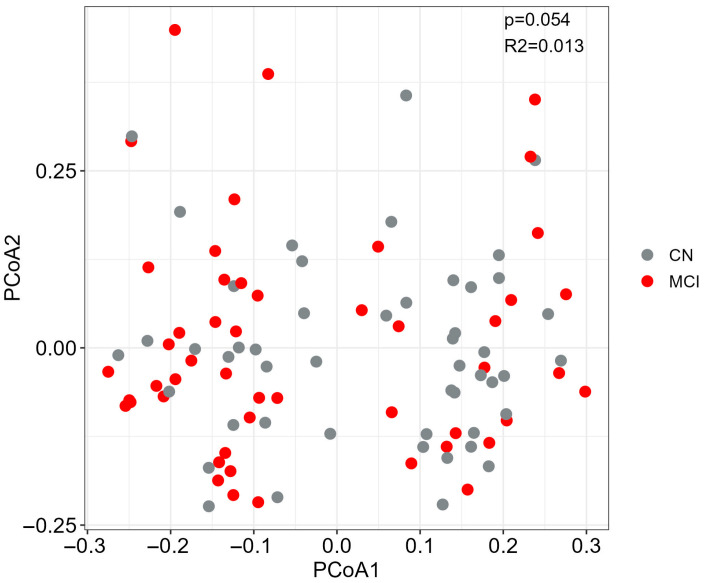
Beta-diversity. Principal coordinate analysis (PCoA) plot of Bray–Curtis dissimilarity distances per clinical diagnosis group was created. *p*-values are from PERMANOVA test (999 permutations), adjusted for selected covariates (Supplementary Text S1). Abbreviations: MCI; mild cognitive impairment, CN: cognitively normal.

We subsequently performed a PERMANOVA analysis to account for confounding factors and to evaluate differences in beta diversity (between-sample diversity). PCoA analysis using Bray–Curtis dissimilarity demonstrated no global differences in the beta diversity by MCI status (R^2^ = 0.013, *p* = 0.054) (Figure 2). Our analysis revealed that sequencing platform had significant effects (PERMANOVA, *q* < 0.05) on the composition of gut microbiota (Figure S3), thus including it as covariate in our differential abundance analysis. Our diversity results are in line with previous studies reporting no differences in alpha and beta diversity indices between healthy controls and individuals with MCI [24,28].

### 3.2. Microbial Alterations in MCI

**Figure 3:**
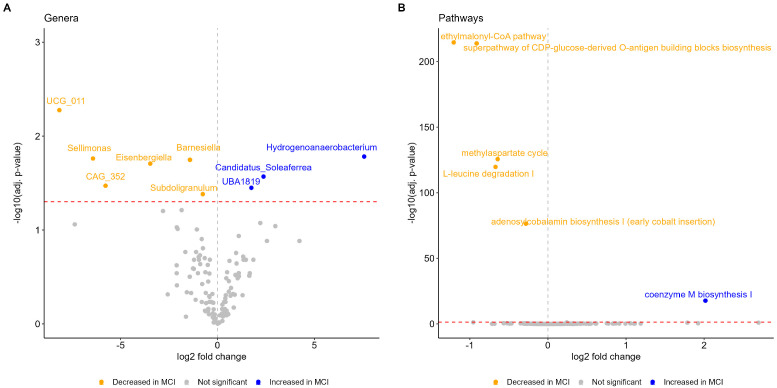
Differentially abundant genera and functional pathways between the cognitively normal and individuals with MCI. Volcano plots display −log10 adjusted *p*-values and log2 fold changes for (**A**) genera and (**B**) pathways between the two groups. Significant changes are highlighted in blue (increased at MCI) or yellow (decreased at MCI) color. Horizontal red dashed line corresponds to adj. *p*-value 0.05 threshold, used to define significant features.

Next, we explored alterations in the gut microbiota at genus level and found significant microbial features associated with MCI status. Using Maaslin2, we identified nine differential genera between the cognitively normal and individuals with MCI (Figure 3A and Table S2), of which eight belong to the Firmicutes phylum and six belong to the families Ruminococcaceae and Lachnospiraceae, which are known for the production of short chain fatty acids (SCFAs). Of note, the direction of effect of these genera was not consistent (e.g., increased *Candidatus_Soleaferrea* in MCI and decreased *Sellimonas* in MCI). Genera most associated with MCI status by magnitude effect included *Hydrogenoanaerobacterium* (log2 fold change; log2FC = 7.56)*, Candidatus_Soleaferrea* (log2FC = 2.37), and *UBA1819* (log2FC = 1.75), while genera most associated with cognitively normal status included *UCG_011* (log2FC = −8.15)*, Sellimonas* (log2FC = −6.42) and *CAG_352* (log2FC = −5.77). We detected altered abundances for genera that, to our knowledge, have not been associated with MCI in humans, to date, although the underlying mechanisms remain unclear and the need for mechanistic validation is emerging.

### 3.3. Functional Profiles Associated with MCI

We found 6 (out of 334 tested) differentially abundant MetaCyc pathways between the cognitively normal and individuals with MCI (Figure 3B and Table S3). Coenzyme M biosynthesis I was the only pathway found with an increased abundance in the MCI group (log2FC = 2.02). Depleted pathways involved ethylmalonyl-CoA pathway (log2FC = −1.21), superpathway of CDP-glucose-derived O-antigen building blocks biosynthesis (log2FC = −0.91), L-leucine degradation I (log2FC = −0.67), methylaspartate cycle (log2FC = −0.64), and the adenosylcobalamin biosynthesis I (early cobalt insertion) (log2FC = −0.28). Coenzyme M biosynthesis I pathway belongs to the methane-producing pathways [53] that are involved in the regulation of microbiota-gut–brain axis and which have been associated with other neurological diseases such as the multiple sclerosis [54]. All depleted pathways have roles in regulating important biological processes. For example, the adenosylcobalamin biosynthesis I pathway supports synthesis of vitamin B12, an essential vitamin not produced by humans, and whose deficiency has been associated with cognitive decline [55]. Our findings regarding functional pathways need to be considered with caution as they cannot infer causality due to 16s rRNA sequencing and further mechanistic studies are needed to clarify the role of these microbial pathways in cognitive decline and MCI.

### 3.4. Correlation of Microbiota with Clinical and Demographic Factors

**Figure 4:**
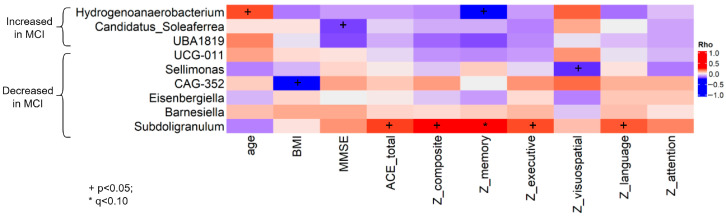
Spearman correlation analysis between differentially abundant genera, clinical and demographic variables. Abbreviations: MCI = Mild Cognitive Impairment; BMI = body mass index; MMSE = mini–mental state examination; ACE = Revised Addenbrooke’s Cognitive Examination.

We calculated Spearman’s correlations between the nine differentially abundant genera, age, BMI, and factors of neurocognitive assessment. We found, in total, 10 significant correlations at a *p*-value < 0.05; however, none passed the adjusted *p*-value < 0.05 cutoff (Figure 4). Half of the correlations involve the genus *Subdoligranulum*, which has been identified at decreased abundance in the MCI group, and which showed correlations with lower ACE and composite z-scores, as well as with three out of five cognitive domains of the z-score (only the correlation with the z-memory score had an adjusted *p*-value < 0.10). Among the genera that were increased in the MCI group, *Candidatus_Soleaferrea* and *Hydrogenoanaerobacterium* showed a negative correlation with MMSE and z-memory score, respectively. *Sellimonas* showed a negative correlation with z-visuospatial score. In regard to demographic data, we identified a positive correlation of *Hydrogenoanaerobacterium* with age and a negative correlation of *Candidatus_Soleaferrea* with BMI.

Of the six Metacyc pathways identified as differentially abundant between the two groups, we identified a positive correlation of Coenzyme M biosynthesis I pathway with z-visuospatial score, without, however, passing the adjusted *p*-value < 0.05 cutoff (Figure S4).

### 3.5. Discriminative Capacity of Microbiota for MCI Status

**Figure 5:**
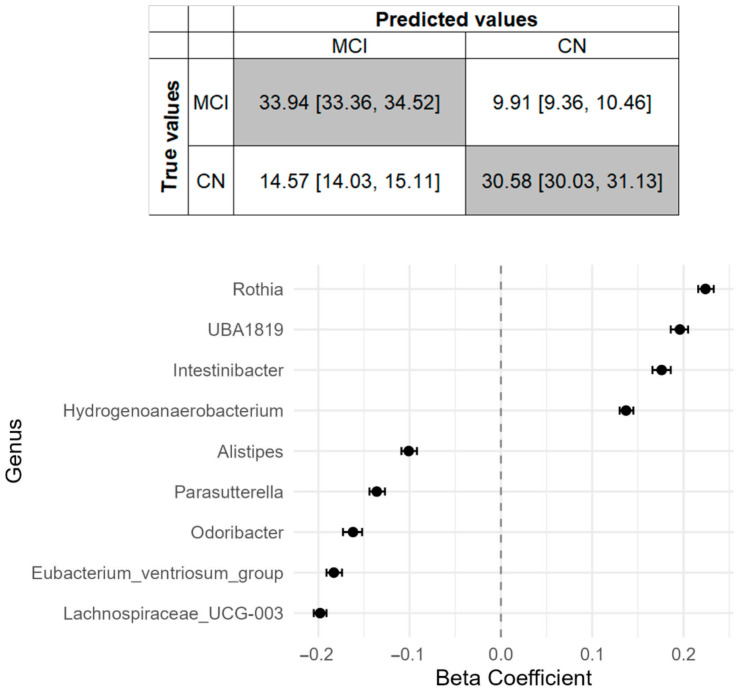
Gut microbiome features associated with MCI based on the discrimination analysis. Confusion matrix obtained from the regularized logistic regression model and genera ranked from the highest to the lowest elastic net positive and negative regression coefficients are shown for the clinically based diagnosis.

The elastic net analysis comparing cognitively normal participants to individuals with MCI identified nine genera discriminating against the MCI status, with *Rothia*, *UBA1819*, *Intestinibacter*, and *Hydrogenoanaerobacterium* over-represented, and *Lachnospiraceae_UCG-003*, *Eubacterium_ventriosum_group*, *Odoribacter*, *Parasutterella* and *Alistipes* under-represented in MCI (Figure 5 and Table S4). The overall predictive accuracy of the classification model (AUC) was 0.77 (95%CI = 0.77–0.78). Our model had moderate diagnostic accuracy based on the confusion matrix, the F1 score (0.73) and the overall prediction accuracy (0.73). Six microbial genera remained in the model after adjusting for covariates, while seven new genera were identified, including the SCFA-producer *Faecalibacterium* (Table S4). Of note, two genera (*Hydrogenoanaerobacterium* and *UBA1819*) were defined as DA genera from Maaslin2 analysis and with the same direction (Figure 3A).

## 4. Discussion

In the present study, we explored gut microbiome profiles associated with MCI in a cohort of 99 individuals without dementia in an aging population sample from Greece. We found microbial genera and metabolic pathways exhibiting significant alterations between the cognitively normal and individuals with MCI. Furthermore, we have highlighted a moderate discriminative capacity of models predicting MCI based on gut microbiota signatures and we have reported significant correlations of microbiome features with cognitive scores.

As shown in previous studies [24,28], our findings that no significant differences in alpha and beta diversity were observed between cognitively normal and individuals with MCI align with earlier research. However, differences in gut microbiome composition and function between healthy and individuals with MCI show large variability across studies [23,24,28,56,57]. We also revealed other microbial genera associated with MCI. Discrepancies among studies may arise from methodological differences in the selected study cohort, sample size, clinical diagnosis criteria for MCI, DNA extraction method, and bioinformatics analysis pipelines, or may be driven by population-specific effects. Most studies exploring gut microbiome profiles in individuals with MCI have been conducted in Asia [24,28,56]. Since, gut microbiome composition and its diversity are heavily influenced by living environment and dietary habits [25], our study, conducted in a European population sample, may help us identify novel microbial signatures associated with MCI. Particularly, in Greece, adherence to the Mediterranean dietary and lifestyle patterns may modulate gut microbiome with potential benefits against the progression of MCI to dementia.

Towards this direction, we identified *Hydrogenoanaerobacterium* among the top associated genera with MCI. In our study, we found this genus at increased abundance in MCI through the DA analysis but also as a discriminative feature for MCI, in a consistent direction, through the elastic net analysis. However, other studies, conducted in populations from Asia [24,28,56], have not observed an association of this genus with MCI, suggesting differences in lifestyle factors, e.g., diet or population-specific driven effects, thus increasing the need to expand microbiome studies in diverse populations with other host and environmental contexts. *Hydrogenoanaerobacterium* functions as a fermenter that can break down various sugars like glucose into metabolites like acetate, ethanol, and hydrogen [58]. Moreover, increased abundance in high fat diet mice has been shown to trigger inflammation by activating the mTOR complex [59], while it has been associated with Parkinson disease [60]. Furthermore, its abundance was negatively associated with Montreal Cognitive Assessment (MoCA) score in individuals with Parkinson’s disease related MCI [61], in line with our study where we highlighted a negative correlation of *Hydrogenoanaerobacterium* with cognitive functioning marked by memory cognitive domain (z-memory score). Toward this direction, we revealed a negative correlation of the MMSE score of cognitive assessment with the MCI-associated *Candidatus Soleaferrea*, a genus with a disease-specific role acting protectively or not [62], in line with a recent work highlighting the role of gut dysbiosis in cognitive decline measured by the MMSE [63]. This is the first study, to our knowledge, reporting an association between these two microbes and cognitive scores; however, further larger studies are needed to elucidate the biological mechanisms beyond this association.

Another interesting observation from the DA analyses is the difference in the directionality of several SCFA-producing microbial genera. SCFAs have numerous roles in health and disease, mitigate the neuroinflammation–neurodegeneration axis and were shown to regulate the maturation and function of the brain [13,64,65]. In our study, SCFA producers that have been associated with the MCI status showed diverse patterns and without having a consistent direction (i.e., some were increased in the MCI and others were decreased). For example, genera belonging to the *Ruminococcaceae* family, known for SCFAs production, showed increased (*Candidatus_Soleaferrea*, *UBA1819*) or decreased (*Subdoligranulum*) abundance in the MCI group, likely reflecting progression of the disease over time, the biological heterogeneity of MCI and the progressive destabilization of gut microbial ecosystems. Notably, the correlation of SCFA producers with cognitive scores are in line with emerging evidence highlighting the potential involvement of microbially produced SCFAs in the microbiota-gut–brain axis and in the AD [66].

Elastic net logistic regression analysis demonstrated a fairly good discriminative capacity of gut microbiota for MCI status (AUC = 0.77), consisting of a signature including nine genera. The highest predictor associated with MCI was *Rothia*, a genus commonly found at the human oral cavity, that has been found to be decreased in the salivary microbiome of AD patients [67] and to be associated with pathological blood biomarkers of AD [68]. Given the recent evidence about the role of oral-microbiota-gut–brain axis in the pathogenesis of neurodegenerative diseases [69] and the contribution of the translocation of oral microbial species into the gut in shaping disease risk [70], our finding moves toward this direction by highlighting the role of *Rothia* in the gut microbiome as important feature for the prediction of MCI; however, larger studies integrating oral and gut microbiome data are needed to substantiate our results. Prediction models play an important role in early disease detection, risk stratification, and personalized care [71]. However, the role of gut microbiome features in predicting MCI status or other prodromal stages of AD continuum is still emerging, with SCFA-producing and pro-inflammatory microbes as key predictors [20,72]. Identifying less-invasive alternatives, compared to existing biomarkers as the pathological markers of AD measured in the cerebrospinal fluid, such as fecal microbiota could improve early screening in prodromal AD stages. Furthermore, identification of specific bacteria reflecting pathophysiological processes will help identify early biomarkers for MCI and will contribute to designing therapeutic strategies to slow rates of cognitive decline. Our results add to the current knowledge suggesting that gut microbiome composition may aid in the detection or in early screening for the disease, though larger studies are necessary to elucidate the underlying biological mechanisms.

To our knowledge, the present study is the first to profile gut microbiome in European individuals with mild cognitive impairment. Current studies focus mainly on Asia [24,28,56], making our study novel and building towards identification of novel microbiome features associated with MCI. However, this study is not without limitations. First, due to the cross-sectional study design, we cannot capture longitudinal effects of gut microbiome changes in AD progression and cannot infer causal associations. However, longitudinal fecal sampling and biomarkers assessments within ALBION study are ongoing (so far up to five years for a few participants), enabling us to identify gut microbiome changes that are associated with the progression of individuals to MCI or symptomatic AD. Second, although we adjusted for covariates in our analyses, we cannot rule out unmeasured and residual confounding effects. For example, dietary habits, with a known role in shaping gut microbiome composition [73], have not been included as covariates. Moreover, potential biases can arise from the use of two different DNA extraction methods and sequencing in two different platforms, although we used sequencing platform as a covariate. Third, our study interrogated gut microbiota profiling through 16s rRNA sequencing, which provides lower taxonomic resolution than shotgun metagenomic sequencing, limiting interpretation. To handle this issue, we inferred functional profiles using PICRUSt2, which, however, depends on the accuracy and completeness of the reference microbiome databases. Finally, we acknowledge that our sample size is modest for microbiome studies not allowing us to perform subgroup analyses, e.g., stratifying participants by *APOEe4* status and might constrain the significance of our results. However, we identified associations at a stringent FDR < 5% threshold. Future studies on a larger scale are required to elucidate the biological mechanisms and to explore microbiome associations with cognitive health.

## 5. Conclusions

In summary, we report gut microbiome alterations in individuals with MCI compared to cognitively normal individuals and we further demonstrate a discriminative capacity of gut microbiota for MCI status. Overall, through the study of an underexplored population of European descent, our findings may facilitate future efforts to determine the role of gut microbiome in the prodromal stages of AD and to identify early markers for AD diagnosis. However, additional research in broader cohorts from different populations is necessary to elucidate the biological mechanisms beyond associations, to assess causal effects and to investigate whether these associations extend to individuals with AD symptoms.

## Figures and Tables

**Table 1:** Participant characteristics.

	CN	MCI	*p*
N (% of total)	49 (49.5)	50 (50.5)	
Female (%)	34 (69.4)	31 (62)	0.4390
Age (yrs)	62.7 (8.6); [42; 84]	68 (9.8); [41; 81]	0.0007
BMI (kg/m^2^)	26.9 (4); [19.8; 37.9]	27.6 (4.9); [14.9; 45.5]	0.3272
*ApoEε4* carriers (%)	13 of 38 genotyped (34.2)	8 of 31 genotyped (25.8)	0.45045
Smokers (%)	16 (32.7)	23 (46)	0.1742
Alcohol consumption (%)	1 (2)	3 (6)	0.3172
Medical history of diabetes (%)	3 (6.1)	8 (16)	0.1179
Medical history of hypertension (%)	11 (22.4)	27 (54)	0.0012
Medical history of dyslipidemia (%)	17 (34.7)	23 (46)	0.2518
Sequencing platform, MiSeq (%)	30 (61.2)	20 (40)	0.0347
Cognitive assessment			
MMSE score	29 (1.5); [22; 30]	27.3 (2); [23; 30]	<0.0001
ACE score	94.9 (3.2); [88; 100]	86.3 (7.67); [61; 99]	<0.0001
Composite Z score	0.2 (0.4); [−0.9; 0.9)	−0.8 (0.9); [−3.8; 0.6)	<0.0001
Z-memory	0.2 (0.6); [−1.3; 1.1]	−1.2 (1.2); [−4.1; 1.2]	<0.0001
Z-attention	0.2 (0.8); [−2.1; 2]	−0.9 (1.3); [−5.8; 1.1]	<0.0001
Z-executive	0.2 (0.6); [−1.6; 1.5]	−0.7 (0.8); [−3.5; 0.5]	<0.0001
Z-language	0.2 (0.5); [−1.1; 1.1]	−0.7 (1.3); [−7.2; 0.5]	<0.0001
Z-visuospatial	0.2 (0.5); [−0.9; 0.7]	−0.5 (2.2); [−12.7; 0.7]	0.0516

Data are shown as n (%) or mean (standard deviation SD) [range]. Fisher’s exact test was used for categorical variables, while *t*-test or Mann–Whitney U test was applied to continuous variables with normal or non-normal distribution, respectively. All variables have complete data apart from BMI (three missing values), MMSE (two missing values), ACE (two missing values), z-scores (two missing values), and *APOEε4* (thirty missing values). Missing data for BMI, MMSE, ACE, and z-scores were imputed by the mean prior statistical analysis. We did not impute for *APOEε4* data. Abbreviations: CN = Cognitively Normal; MCI = Mild Cognitive Impairment; BMI = body mass index; MMSE = mini-mental state examination; ACE = Revised Addenbrooke’s Cognitive Examination.

## Data Availability

All data used in the analyses of this study are available within the manuscript and its Supplemental Information files. The raw sequencing data generated from this study have been deposited in NCBI SRA (https://www.ncbi.nlm.nih.gov/sra, assessed on 7 September 2025) under the accession number PRJNA1297934.

## Supplementary Materials

The following supporting information can be downloaded at: https://www.mdpi.com/article/10.3390/microorganisms13092112/s1, Figure S1: Schematic of the study’s workflow. Figure S2: Compositional plots of relative abundance among the clinically based diagnosis groups at phylum, family and genus level; Figure S3: Effect of sequencing platform on the gut microbiome composition; Figure S4: Spearman correlation analysis between differentially abundant pathways, clinical and demographic variables; Supplementary Text S1: Selection of covariates for DA analysis models; Supplementary Text S2: Discrimination analysis; Table S1. Alpha-diversity metrics in the clinically based diagnosis subgroups; Table S2: Maaslin2 results for the identification of differentially abundant genera between cognitively normal and individuals with MCI; Table S3: Maaslin2 results for the identification of differentially abundant pathways between cognitively normal and individuals with MCI; Table S4: List of microbiota taxa selected 100% times in the 10-fold cross-validation for cognitively normal vs. individuals with MCI.

## Abbreviations

The following abbreviations are used in this manuscript:

AD	Alzheimer’s disease
CSF	Cerebrospinal fluid
MCI	Mild cognitive impairment
CN	Cognitively normal
BMI	Body mass index
