# Supplementary material for: Gut Microbiome Alterations in Mild Cognitive Impairment: Findings from the ALBION Greek Cohort
Source: Microorganisms. 2025 Sep 10;13(9):2112. doi: 10.3390/microorganisms13092112 (PMC12473069; doi:10.3390/microorganisms13092112)
Supplement: Supplementary file 1 [file microorganisms-13-02112-s001.zip › microorganisms-3817243_supplement_revised.pdf]

Supplementary Materials

Supplementary Text S1. Selection of covariates for DA analysis models

For the differential analysis using Maaslin2, we ran ZINB and NEGBIN including covariates as fixed effects. Covariates were selected based on their difference between subgroups (**Table 1**) and on their effects on the gut microbiome composition as revealed from the PERMANOVA analysis. The following covariates were used: age, sex, medical history of hypertension, sequencing platform, MMSE.

Supplementary Text S2. Discrimination analysis

Genera associated with classification based on clinical diagnosis

To identify a gut microbiota signature for MCI, we regressed diagnosis status (MCI vs cognitively normal) on the 139 genera using a logistic regression with elastic net penalty. To reduce overfitting, the data was divided into training set (90%) and test set (10%). In the training set, we evaluated the alpha parameter from 0.1 to 1 in ~0.05 increments (avoiding L2 norm), and the tuning parameter (lambda), using a leave-one-out cross-validation (given the small sample size) approach in a 30-iteration loop. The combination of alpha and lambda was chosen based on a high Area Under the ROC Curve (AUC) in the test set. We found that alpha = 0.20 and lambda = 0.13919207 gave the highest performance in the test set. We applied the selected alpha and lambda values to each penalized regression for every training (90% of our primary dataset) in a 10-iteration loop. The coefficients obtained from 10 iterations in the elastic net were applied to the selected genera as weights (positive or negative) to estimate the signature of MCI as the weighted sum. For each genus, we calculated the mean coefficient and the 95% CI.

To address potential confounding effects of age, sex, medical history of hypertension, sequencing platform and MMSE, we added them in the elastic net model as covariates (unpenalized).

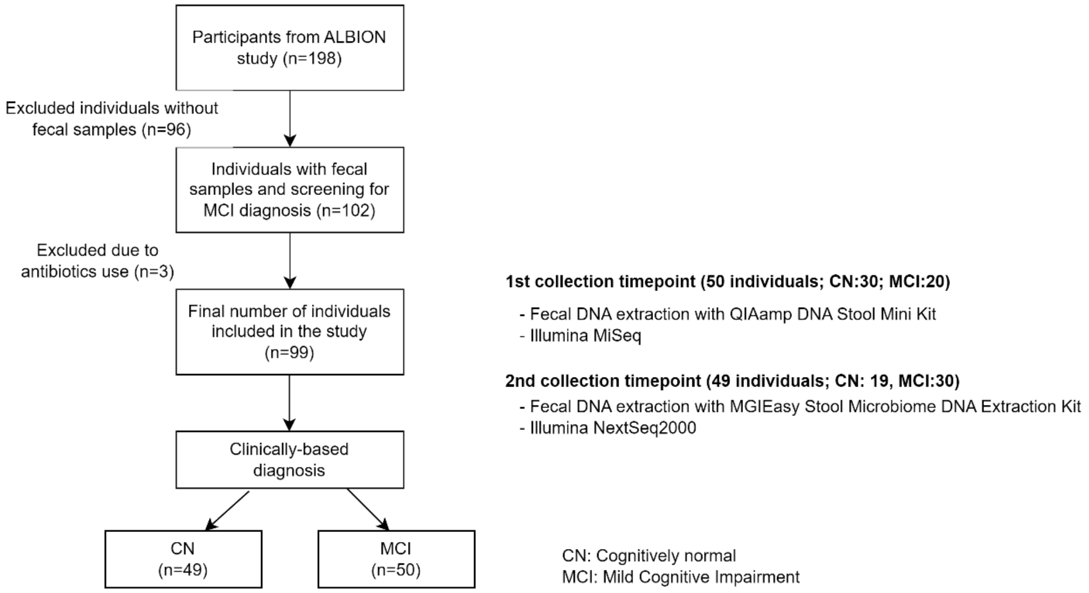

Figure S1. Schematic of the study's workflow.

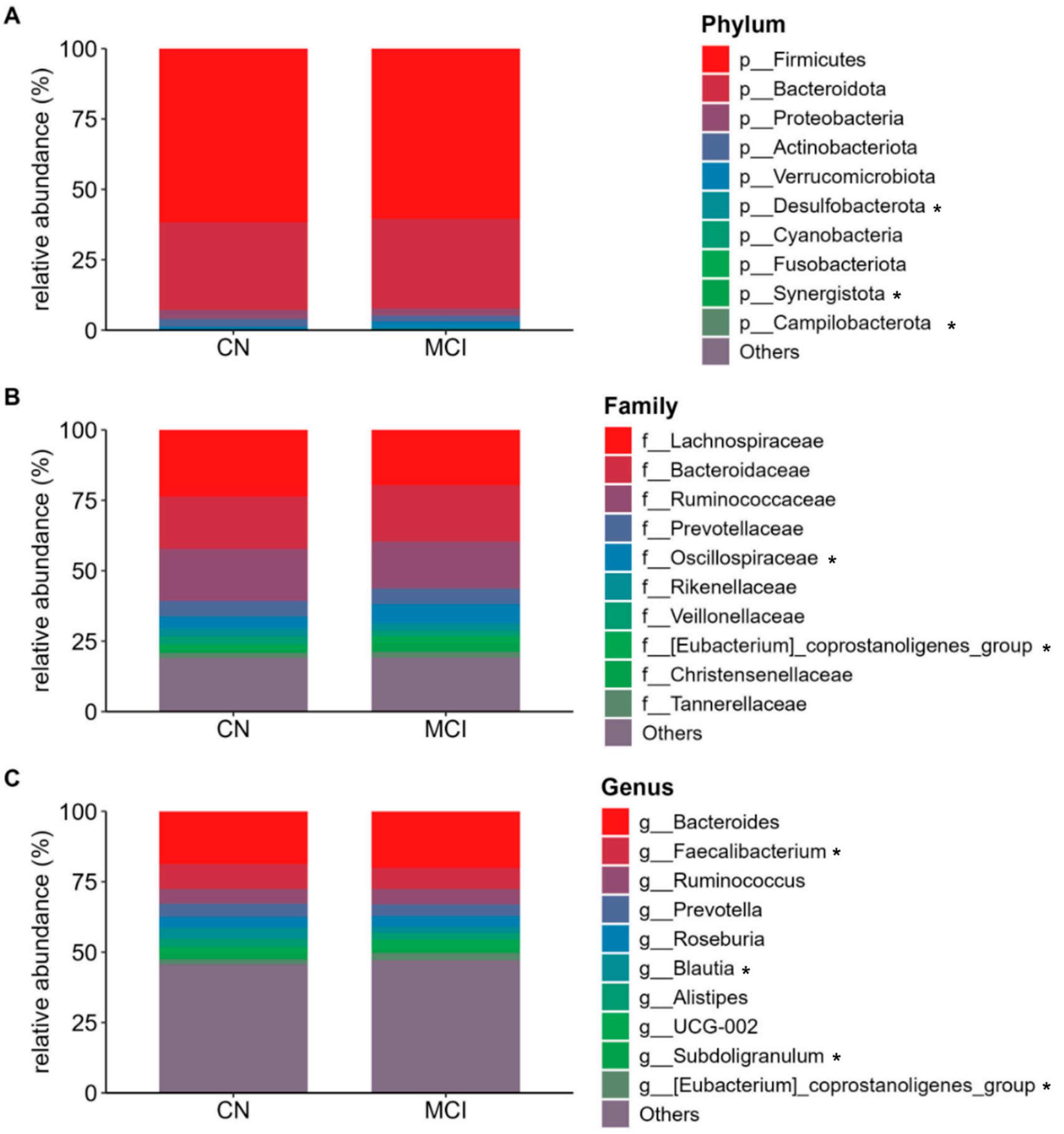

Figure S2. Compositional plots of relative abundance among the clinically-based diagnosis groups at phylum, family and genus level. Taxa with an asterisk (*) showed a statistically significant difference between groups based on Wilcoxon test. In the taxa labels, *'p'* refers to phylum, *'f'* refers to family and *'g'* refers to genus. Genera shown in Panel C belong to phyla as following: *Bacteroides* (Bacteroidota); *Faecalibacterium* (Firmicutes); *Ruminococcus* (Firmicutes); *Prevotella* (Bacteroidota); Roseburia (Firmicutes); *Blautia* (Firmicutes); *Alistipes* (Bacteroidota); *UCG-002* (Firmicutes); *Subdoligranulum* (Firmicutes); *[Eubacterium]_coprostanoligenes_group* (Firmicutes).

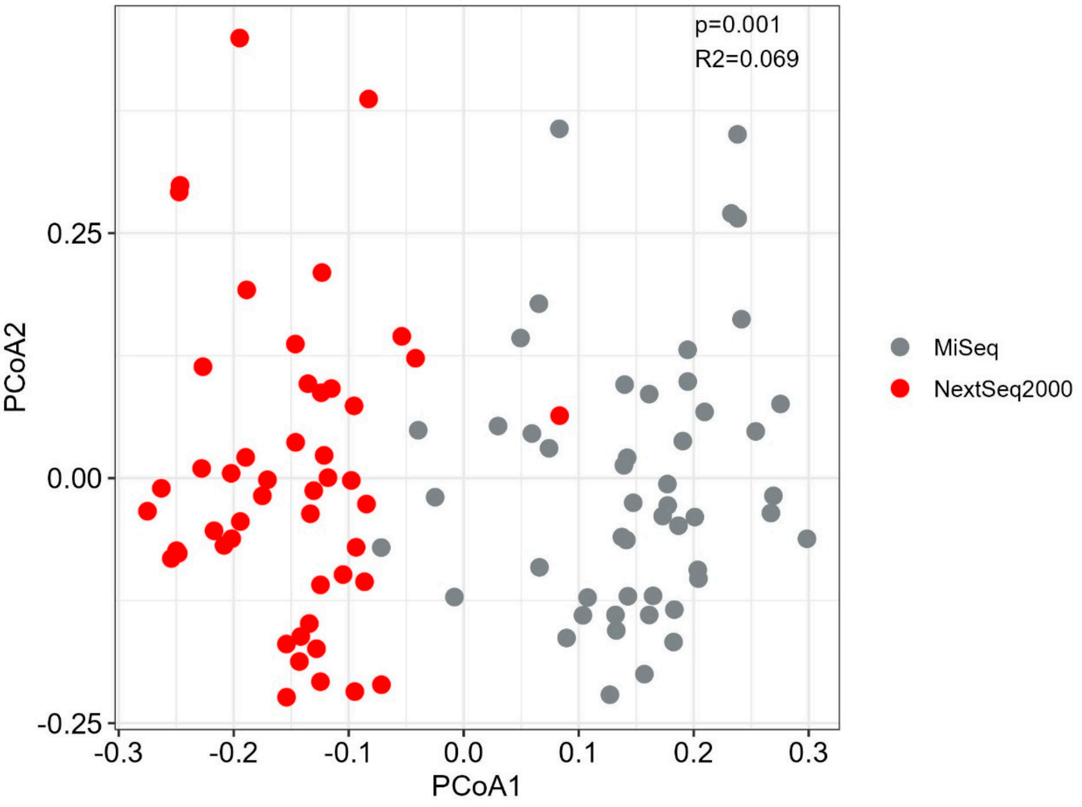

Figure S3. Effect of sequencing platform on the gut microbiome composition. P-value is from PERMANOVA (999 permutations).

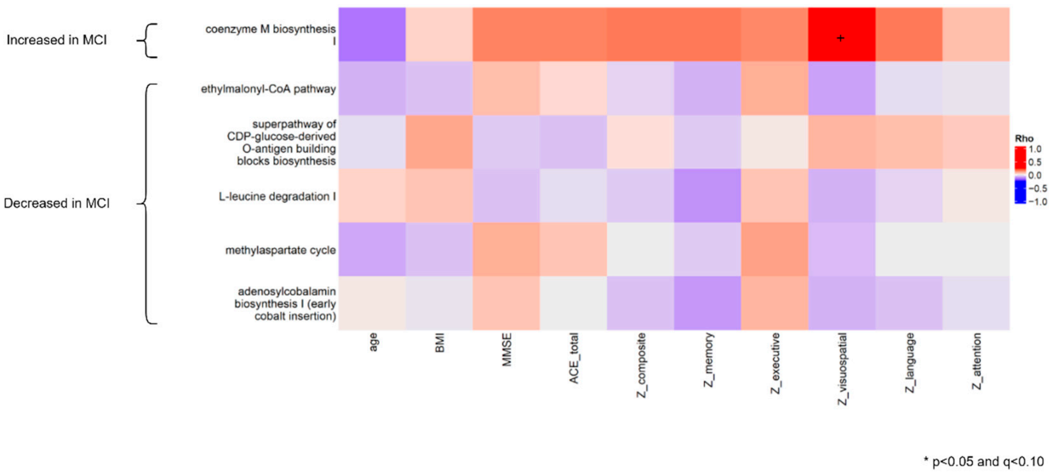

Figure S4. Spearman correlation analysis between differentially abundant pathways, clinical and demographic variables.
